# Bifidobacterium: An Emerging Clinically Significant Metronidazole-resistant Anaerobe of Mixed Pyogenic Infections

**DOI:** 10.7759/cureus.1134

**Published:** 2017-04-04

**Authors:** Hena Butta, Raman Sardana, Raju Vaishya, Kailash N Singh, Leena Mendiratta

**Affiliations:** 1 Microbiology, Indraprastha Apollo Hospitals; 2 Orthopaedics, Indraprastha Apollo Hospitals; 3 Nephrology, Indraprastha Apollo Hospitals

**Keywords:** bifidobacterium, metronidazole resistant, pyogenic infection

## Abstract

*Bifidobacterium *sp. are anaerobic, Gram-positive, short, irregular rods with rudimentary branching. These organisms are reported to be associated with health benefits and their significance as a pathogen is not much reported. We are reporting two cases of mixed pyogenic infections due to *Bifidobacterium* sp. In the first patient, the infection mimicked tubercular bone infection. The second patient was a case of hydronephrosis with double J (DJ) stent blockage. In both cases, *Bifidobacterium* sp. was isolated in combination with *Escherichia coli* from the evacuated pus samples. The factors which contributed significantly for detecting *Bifidobacterium* sp. were gram stain examination, use of rapid and automatic anaerobic cultivation system (Anoxomat, MART Microbiology B.V., The Netherlands), and quick identification by MALDI-TOF (Matrix-Assisted Laser Desorption-Time-of-Flight) Mass Spectrometry (Biomerieux, France). Both strains were found to be resistant to metronidazole and both patients showed a good clinical response to treatment with beta-lactam antibiotics. So, we highlight the importance of seeking *Bifidobacterium* species in all clinical pyogenic samples.

## Introduction

*Bifidobacterium* sp. are anaerobic, non-motile, non-acid-fast, Gram-positive, short, irregular rods. They appear as bifid or irregular V or Y-shaped rods with rudimentary branching. They belong to phylum *Actinobacteria*, order *Bifidobacteriales,* and family *Bifidobacteriaceae* [1]. These constitute the normal flora of the gastrointestinal tract and the oral cavity and are not frequently isolated from human clinical specimens. There are many species in genus *Bifidobacterium*, and a few of these have been implicated in health benefits like prevention of diarrhea, the establishment of a healthy microflora in premature infants, reducing colonic transit time, lactose intolerance, reducing serum cholesterol levels, immunomodulatory effects, and even in the prevention of gastrointestinal cancers [1]. Thus, these strains of *Bifidobacterium* sp. are also being used as probiotics for the treatment of gastrointestinal tract infections and antibiotic-associated diarrhea. The reports implicating *Bifidobacterium* sp. as a pathogen for human beings are scarce. The significant infections due to *Bifidobacterium* sp. have been documented from abdominal abscesses, obstetric/gynecological sites, and wounds [2]. We are reporting two cases of mixed pyogenic infections due to *Bifidobacterium* sp.

## Case presentation

### Case 1

**Figure 1:**
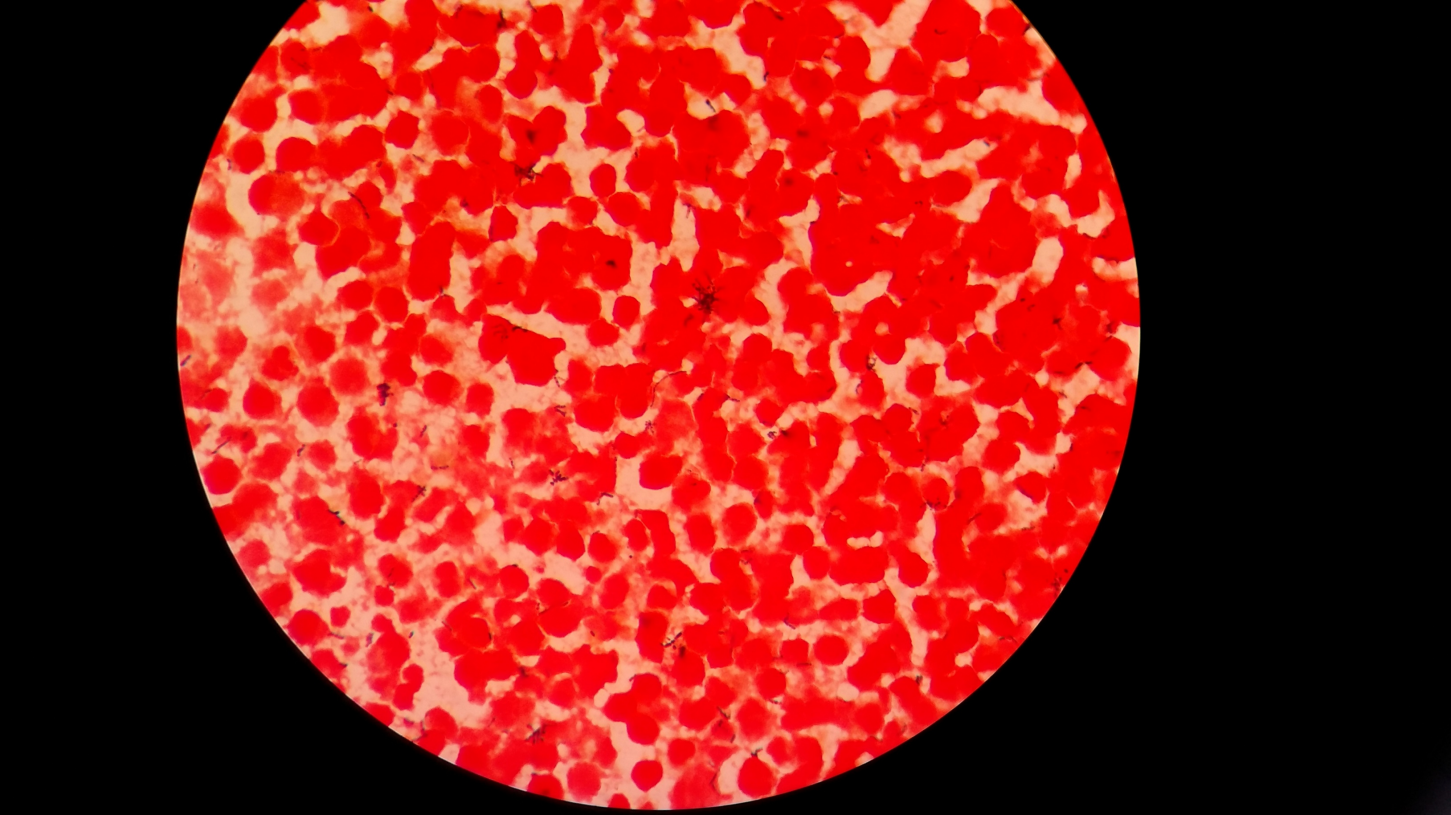
Gram stain examination (1000 X)- showing unevenly stained irregular Gram-positive coccobacillary organisms with rudimentary branching

A 38 year-old-male presented in July 2016 with complaints of pain over the right hip joint for 10 days and intermittent fever for three months. The patient was apparently well prior to the three months. He subsequently developed pain in the lower back, which was insidious in onset, gradually progressive, and associated with intermittent fever. MRI findings were suggestive of bilateral tubercular sacroiliitis and multifocal intramuscular bone collection. Therefore, the patient was placed on antitubercular treatment for two months but with no clinical response. On local examination, a diffuse swelling was present in the right lower limb. The swelling was tender with pitting edema. The hip and knee were fixed in flexion, 30° and 90°, respectively. The diagnosis of bilateral sacroiliitis, psoas abscess of the right side, and right thigh and gluteal region abscess was made. Incision and drainage of the right hip joint were done, and about 500 ml of purulent material was evacuated. The patient was empirically started on ceftriaxone and amikacin. On Gram stain examination of the pus, plenty of unevenly stained Gram-positive coccobacillary organisms and occasional Gram-negative bacilli were seen (Figure 1). Zeihl Neelsen staining for AFB (acid-fast bacilli) was negative. 

For anaerobic culture, the sample was inoculated on brain heart infusion agar, Columbia blood agar and phenylethyl alcohol agar and anaerobiosis was generated by using Anoxomat. After 48 hours of incubation, two types of colonies seen on the culture were Gram stained and aerotolerance was done. The anaerobic bacterium was identified as *Bifidobacterium* sp. by both MALDI-TOF Vitek® MS (Mass Spectrometry) and Vitek® 2 using ANC card (bioMérieux, Marcy l'Etoile, France). The aerotolerant colonies were identified as *Escherichia* coli, which were also grown in aerobic culture. *Escherichia coli *was found to be extended spectrum beta-lactamase (ESBL)-positive and susceptible to amoxicillin + clavulanic acid, piperacillin + tazobactam, cefoperazone + sulbactam, imipenem, meropenem, amikacin, and chloramphenicol. Bifidobacterium sp. was found to be sensitive to imipenem, meropenem, amoxicillin + clavulanic acid, piperacillin + tazobactam, and clindamycin but it was resistant to metronidazole. After the culture and sensitivity report, the patient was administered piperacillin + tazobactam for 15 days followed by amoxicillin + clavulanic acid. AFB culture by BACTEC™ MGIT™ 960 system (Becton, Dickinson & Co., Franklin Lakes, NJ) remained negative after six weeks of incubation. The patient responded well to the treatment and recovered fully after three months of antibiotic therapy.

### Case 2

**Figure 2:**
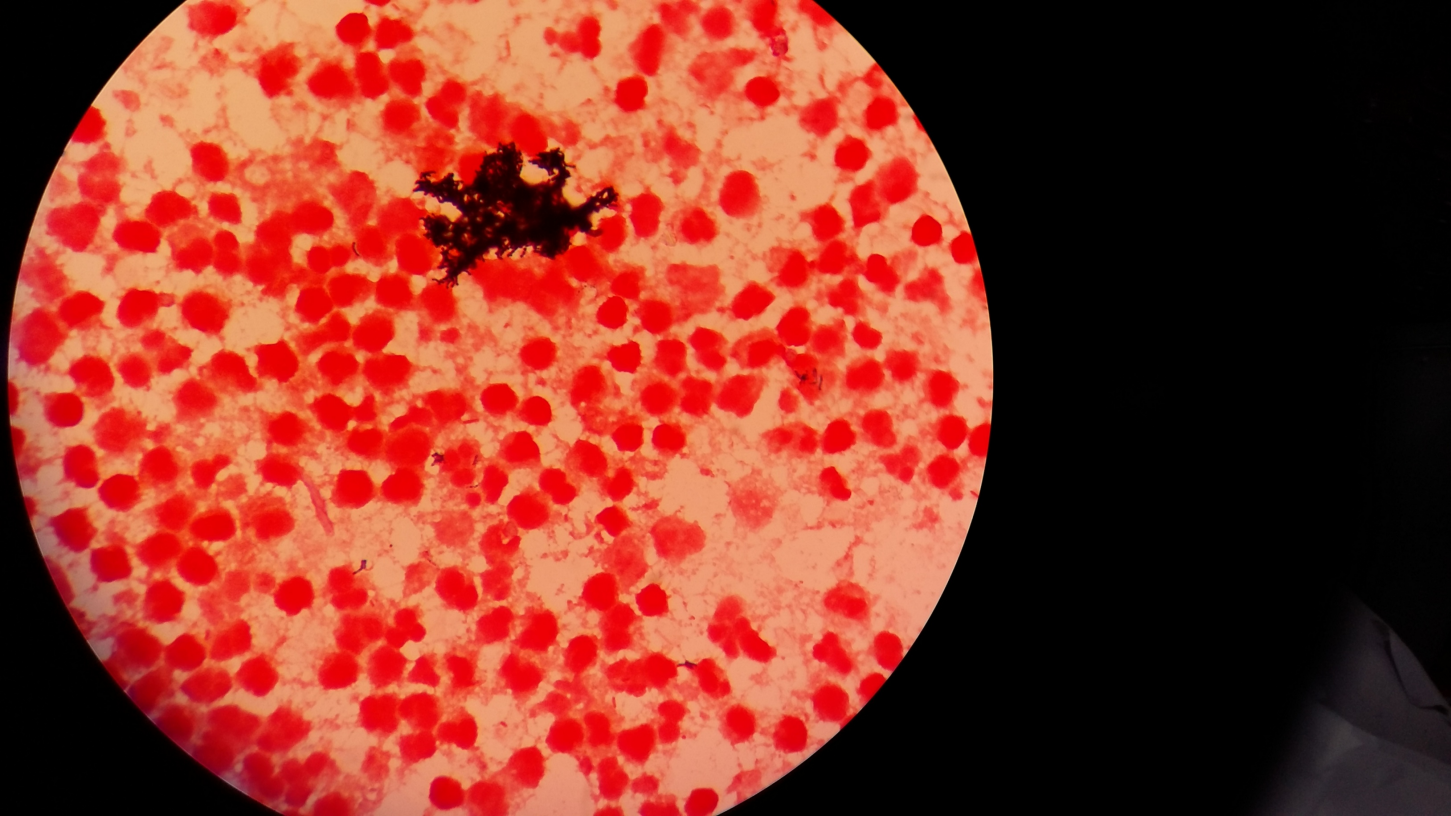
Gram stain examination (1000 X)- showing irregular, branching Gram-positive bacilli

A 51-year-old female presented in September 2016 with decreased urine output, generalized weakness, and decreased appetite. The patient was a known case of Type 2 diabetes mellitus for two years, hypertension for 30 years, cervical carcinoma (post-chemotherapy and radiotherapy, 22 cycles) diagnosed in 2012 with recurrence in 2015, and obstructive nephropathy with double J (DJ) stent in-situ on the right side since January 2016. On examination, the patient was afebrile with other systems within normal limits. Her total leucocyte count (TLC) was 12,400/mm3, blood urea was 69 mg/dl, serum creatinine was 3.7 mg/dl, and potassium was 5.1 meq/l.  Liver function tests were within normal limits. Abdominal ultrasound showed moderate hydronephrosis of the right kidney and increased parenchymal echogenicity with diminished corticomedullary differentiation of the left kidney. Computed tomogram (CT) scan of the abdomen showed right kidney hydroureteronephrosis with DJ stent in situ, and the left kidney showed parenchymal atrophy with irregular margins. The patient was diagnosed as a case of acute chronic kidney disease with DJ stent blockage. The DJ stent was changed, and the pus was evacuated. Gram stain examination of the pus showed plenty of branching Gram-positive bacilli, along with Gram-negative bacilli (Figure 2).  

The specimen was processed similarly to Case 1. Escherichia coli was grown in aerobic culture, and *Bifidobacterium* sp. was cultured in anaerobic culture. The identification of *Bifidobacterium* sp. was done by both MALDI-TOF Vitek® MS and Vitek® 2. *Escherichia coli *was found to be extended spectrum beta-lactamase (ESBL)-positive and sensitive to piperacillin + tazobactam, cefoperazone + sulbactam, imipenem, meropenem, amikacin, gentamicin, tobramycin, chloramphenicol, and co-trimoxazole. *Bifidobacterium* sp. was found to be sensitive to penicillin, ceftriaxone, imipenem, meropenem, amoxicillin + clavulanic acid, piperacillin + tazobactam, and clindamycin and was resistant to metronidazole. The patient showed a good response to meropenem and recovered completely.

## Discussion

*Bifidobacterium* sp. has been studied in disease association with altered gut microbiota. The various species of *Bifidobacterium* that have been used as probiotics are *B. bifidum*, *B. breve*, *B. lactis*, *B. longum*, *B. infantis, *and *B. adolescentis *[3]. It is important to note that not all the species of *Bifidobacterium* are beneficial. Also, the species which have been used as probiotics have also been reported to cause infections, especially in neonates and immunocompromised individuals (See Table 1). The case reports of *Bifidobacterium* sp. as significant pathogens are listed in Table 1.

**Table 1:** Cases of Bifidobacterium sp. - Review of the Literature

Year	Age/Sex	Site of infection	Species of *Bifidobacterium*	Immune status
2016 Esaiassen, et al. [4]	Three preterm infants (two males and one female)	Blood	*Bifidobacterium longum subsp. infantis*	Preterm infants
2015 Weber, et al. [5]	74 years/Male	Blood	*Bifidobacterium longum*	On chemotherapy, radiotherapy for polymetastatic prostatic adenocarcinoma
2015 Weber, et al. [5]	21 cases of bacteremia due to *Bifidobacterium* sp. reviewed			
2015 Avcin, et al. [6]	2 years/ Male	Blood	*Bifidobacterium breve*	Acute lymphoblastic leukemia on chemotherapy
2014 Suwantarat, et al. [7]	45 Years/Female	Ventriculoperitoneal shunt	*Bifidobacterium breve*	Cerebral palsy, congenital hydrocephalus, VP shunt placement since two months of age with latest revision two years previously.
2014 Pathak, et al. [8]	66 years/Female	Urinary tract	*Bifidobacterium species*	Myelodysplastic syndrome, hepatitis C, cirrhosis, uncontrolled diabetes
2012 Barberis, et al. [9]	80 yrs/Female	Urinary tract	*Bifidobacterium scardovii*	Breast cancer treated with chemotherapy and radiotherapy, autoimmune haemolytic anaemia, on steroids

It has been seen that *Bifidobacterium* sp. is associated with good oral health. On the other hand, it has also been found to be a predominant microbe in dental caries, and *Bifidobacterium *dentiumis strongly linked to dental caries. *Bifidobacterium* sp. has also been reported in cases of meningitis [10]. Nevertheless, the reports of *Bifidobacterium* sp. association as a significant human pathogenic agent are less. *Bifidobacterium* sp. can cause significant infections, and the presentation of the case can also mimic tubercular infection, such as the patient in our case. The scarcity of the clinical cases can be because of their fastidious nature, a special requirement of anaerobic atmosphere and, thus, difficult isolation. The isolation of *Bifidobacterium* sp. can be improved by the direct microscopic examination, i.e. Gram stain of the clinical specimen, appropriate anaerobic techniques, and a rapid and accurate identification system. In the present reports, *Bifidobacterium* species were isolated in combination with *Escherichia coli*. If the Gram stain examination had not been done, it could have been overlooked. Thus, the Gram stain examination gave us the initial clue of such a type of bacteria. The anaerobic atmosphere was generated by the more stringent Anoxomat™ technique and the identification was made by MALDI-TOF Vitek® MS system. Moreover, the identification by the Vitek® 2 system also corroborated with MALDI-TOF system. In addition to above factors, the underreporting of *Bifidobacterium* sp. can be due to its presence in combination with other bacteria at the site of infection, resistance to metronidazole, and susceptibility to beta-lactam drugs, which are commonly used for the treatment of common aerobic Gram-negative bacteria.

## Conclusions

*Bifidobacterium* sp. is emerging as an important causative agent of pyogenic infections, either alone or in combination with other aerobic bacteria (mixed infection). The infections due to these bacteria are underreported due to their fastidious nature, the requirement of strict anaerobic conditions to grow, difficult identification methods, and their susceptibility to beta-lactam drugs, which are commonly used for the treatment of aerobic bacteria. Also, there are a few species of *Bifidobacterium* that are used for health benefits. Therefore, these bacteria have not drawn the attention of microbiologists and clinicians for their association as a significant pathogen. Our case reports reveal *Bifidobacterium *sp. as an important cause of pyogenic infection and their clinical presentation mimicking the tubercular infection also in an otherwise immunocompetent patient. The direct microscopic examination is crucial for getting the initial clue of infection due to *Bifidobacterium* sp., and thus, it helps in deciding appropriate culture media and incubation conditions for the cultivation of the organism. Also, beta-lactam drugs are losing their importance as frontline drugs and metronidazole would not cover these bacteria for treatment. So, we highlight the importance of seeking them in all clinical pyogenic specimens.
